# High-Dose Ceftriaxone in Elderly Patients with Enterococcal Infective Endocarditis: Population Pharmacokinetics of Free Ceftriaxone and Dose Optimization

**DOI:** 10.3390/antibiotics14050508

**Published:** 2025-05-15

**Authors:** Beatriz Fernández Rubio, Fernando Docobo Pérez, Laura Herrera Hidalgo, Luis Eduardo López-Cortés, Rafael Luque Márquez, José Manuel Lomas Cabezas, Luis Fernando López-Cortés, Marta Mejías Trueba, Ana Belén Guisado Gil, Alicia Gutiérrez Valencia, Arístides de Alarcón González, María Victoria Gil Navarro

**Affiliations:** 1 Unidad de Gestión Clínica de Farmacia, Instituto de Biomedicina de Sevilla (IBiS), Hospital Universitario Virgen del Rocío/CSIC/Universidad de Sevilla, 41013 Seville, Spain; 2 Departamentos de Medicina y Microbiología, Universidad de Sevilla, 41004 Seville, Spain; 3 Unidad Clínica de Enfermedades Infecciosas y Microbiología, Hospital Universitario Virgen Macarena, Instituto de Biomedicina de Sevilla (IBiS)/CSIC, Universidad de Sevilla, 41004 Seville, Spain; 4 Unidad Clínica de Enfermedades Infecciosas, Microbiología y Parasitología (UCEIMP), Instituto de Biomedicina de Sevilla (IBiS), Hospital Universitario Virgen del Rocío/CSIC/Universidad de Sevilla, 41013 Seville, Spain; 5 CIBER de Enfermedades Infecciosas, Instituto de Salud Carlos III, 28029 Madrid, Spain; 6 Primary Care Pharmacist Service, Sevilla Primary Care District, 41018 Seville, Spain

**Keywords:** ceftriaxone, pharmacokinetics, elderly patients, Monte Carlo simulations

## Abstract

**Background:** Ampicillin plus ceftriaxone (AC) is a first-line treatment for *Enterococcus faecalis* infective endocarditis (IE). Its administration in outpatient parenteral antibiotic treatment (OPAT) programs is challenging. The design of a ceftriaxone regimen suitable for OPAT requires deep knowledge of ceftriaxone pharmacokinetics (PK). **Objective:** We aim to explore ceftriaxone PK in elderly patients and propose dose regimens adapted to OPAT to maintain synergistic concentrations (Cs) with ampicillin against *E. faecalis*. **Methods:** We conducted a prospective observational pharmacokinetic study on patients (>55 years old) affected by *E. faecalis* IE. Ceftriaxone free concentration was measured at three time-points: before the administration (C_min_) and two and four hours after ceftriaxone administration (C_2_ and C_4_). Both structural and covariate population pharmacokinetic models were built. Monte Carlo simulations of six ceftriaxone dosages were performed and the probability of target attainment (PTA) of an optimal Cs range was analyzed. The pharmacokinetic/pharmacodynamic index (PK/PD) to predict efficacy was defined as maintaining free ceftriaxone concentrations superior to the Cs at 50–100% of the dosing interval (fT ≥ Cs ≥ 50–100% of the dosing interval). Ceftriaxone dosing regimens were considered optimal if at least 90% of the simulated population was able to achieve the defined PK/PD targets. **Results:** Twenty-four episodes from 16 patients were included. Mean free ceftriaxone concentration pre-dose, +2 h, and +4 h were C_min_ = 7.8 ± 6.5 mg/L, C_2_ = 34 ± 26.5 mg/L, and C_4_ = 22.7 ± 19.7 mg/L, respectively. A two-compartment model with first-order absorption and elimination best described the data. Ceftriaxone one-hour infusions only achieved the minimum PK/PD target when the 2 g/12 h regimen was tested. On the other hand, ceftriaxone continuous infusion maintained a Cs above the PK/PD target for 100% of the dosing interval using ceftriaxone 4–6 g regimens. **Conclusions:** Our findings suggest that the optimal ceftriaxone exposure may be achieved using high-dose continuous infusions to ensure an ampicillin-killing effect when treating *E. faecalis* IE.

## 1. Background

Infective endocarditis (IE) is a life-threatening disease whose epidemiological profile has evolved over the past decade [1]. Nowadays, *Enterococcus* spp. is the third worldwide leading cause of IE (10–15% of all IE), with *Enterococcus faecalis* being the responsible microorganism in 90% of the cases [2,3]. Patients affected by *E. faecalis* IE are characterized by an advanced age, a large burden of comorbidities, high rates of cancer, aortic valve affectation and, sometimes, a previous history of urinary tract or abdominal infections [4,5]. The source of acquisition is mainly associated with healthcare [2,5,6]; as an example, *E. faecalis* is the first leading cause of IE in transcatheter aortic valve implantation (TAVI) [7].

First-line antibiotic regimens for *E. faecalis* IE comprise the combination of high-dose penicillin (ampicillin, amoxicillin, or benzylpenicillin) plus a synergistic agent (ceftriaxone or gentamicin) for 4–6 weeks [8,9]. Ampicillin combined with high-dose ceftriaxone (AC) is the preferred treatment for *E. faecalis* IE when treating elderly and fragile patients because of this combination has demonstrated high efficacy rates and better safety profile than the alternatives [10]. To avoid long inpatient treatments, the inclusion of patients with IE in outpatient parenteral antibiotic treatment programs (OPAT) has been endorsed worldwide [11,12,13,14]. The inclusion of first-line treatments for *E. faecalis* IE in OPAT is challenging, because these regimens include combined therapy administered multiple times per day [4].

Some aspects might be considered when designing an AC outpatient regimen. AC is a synergistic combination, where the active agent is ampicillin, and ceftriaxone potentiates its activity [15,16]. The pharmacokinetic/pharmacodynamic index (PK/PD) correlated with maximal bacterial killing activity of antimicrobial combinations is poorly defined [17,18]. In this case, the main goal is to maintain synergistic concentrations (Cs) of both antibiotics, which accounts for ampicillin concentration above the minimum inhibitory concentration (MIC) of the microorganism and free ceftriaxone concentration above the 5–10 mg/mL, in both cases for at least 50% of the dosing interval [18,19]. The transition to OPAT of a multidose combined regimen (ampicillin 2 g every 4 h and ceftriaxone 2 g every 12 h) demanded a reduction in drug administrations for a good compliance. In this regard, AC continuous infusion has been proposed as an alternative in OPAT to the inpatient regimen [20].

Ceftriaxone pharmacokinetics has been widely studied, highlighting that patient characteristics such as age, comorbidities and fragility could potentially lead to significant changes in PK/PD. Typical PK values for ceftriaxone has been previously described (half-life = 7.5–8.9 h, plasma protein binding = 79–94%, volume of distribution = 11.01–10.69 L, clearance = 833–1023 mL/h). Ceftriaxone plasma protein binding is high in healthy volunteers and young individuals; however, hypoproteinemia might lead to unexpected high free ceftriaxone concentrations in geriatric patients [21,22]. In the present study, we aim to explore the PK of ceftriaxone in elderly patients treated with high-dose ceftriaxone regimens and the potential for various dose regimens adapted to OPAT to maintain the necessary synergistic concentrations with ampicillin against *E. faecalis.*

## 2. Results

### 2.1. Patients and Ceftriaxone Concentrations

Twenty-four episodes from 16 patients were included in the study. Ten patients (63%) were males, accounting for 18 episodes (75%). Demographic, clinical and microbiological variables are shown in Table 1. Mean total ceftriaxone concentration pre-dose, +2 h, and +4 h were C_min_ = 51.6 ± 21.9 mg/L, C_2_ = 129 ± 53.2 mg/L, C_4_ = 105.2 ± 49.4 mg/L, respectively. Mean free ceftriaxone concentration pre-dose, +2 h, and +4 h were C_min_ = 7.8 ± 6.5 mg/L, C_2_ = 34 ± 26.5 mg/L, C_4_ = 22.7 ± 19.7 mg/L, respectively. Mean ceftriaxone plasma protein binding (PPB) at different time-points were 85.7 ± 6.4% pre-dose, 74.7 ± 10.1% 2 h after its administration, and 79.6 ± 9.3% 4 h after its administration.

### 2.2. Pharmacokinetic Model

A two-compartment model with first-order absorption and elimination best described the data for free ceftriaxone. The PK parameters of the model were clearance (CL), central volume (V_1_), intercompartmental clearance (Q), and peripheral volume (V_2_) (Table 2).

Creatinine clearance (CrCl) and body mass index (BMI) were related to drug clearance (Cl) and central volume of distribution (V_1_), respectively, using a log-linear model, as follows:

$${Cl}_{i}={Cl}_{pop}\times \left(\frac{{Cl}_{i}}{59.5}\right)\beta_{CrCl}$$

$$V_{1i}=V_{1pop}\times \left(\frac{V_{i}}{29.3}\right)\beta_{BMI}$$

**Figure 1:**
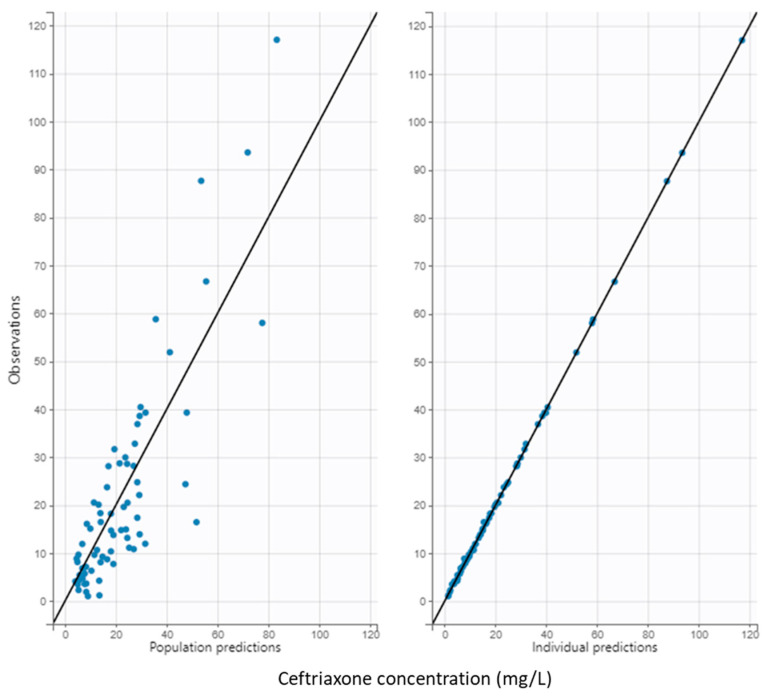

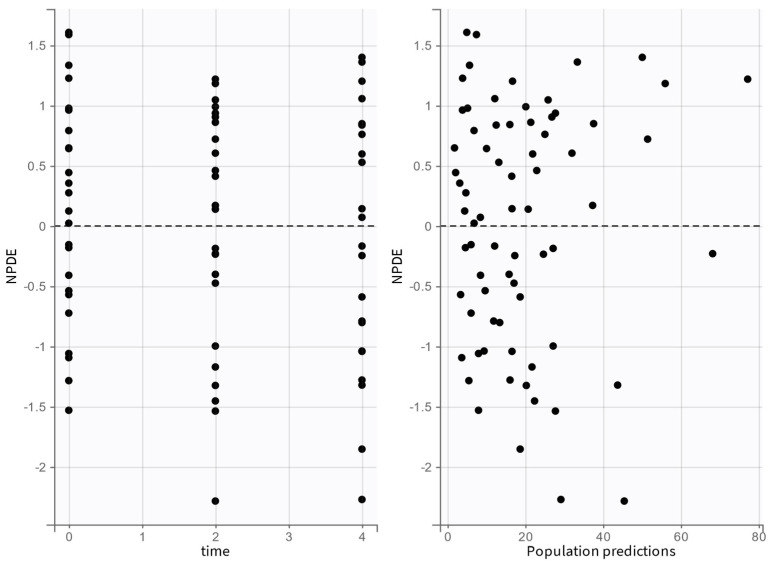
Goodness-of-fit plots from the final model. Black dots represent unbound ceftriaxone concentrations. NPDE is the normalized prediction distribution error. The lines represent identity lines (solid, **top plots**) or the theoretical mean of NPDE (dashed, **bottom plots**).

**Figure 2:**
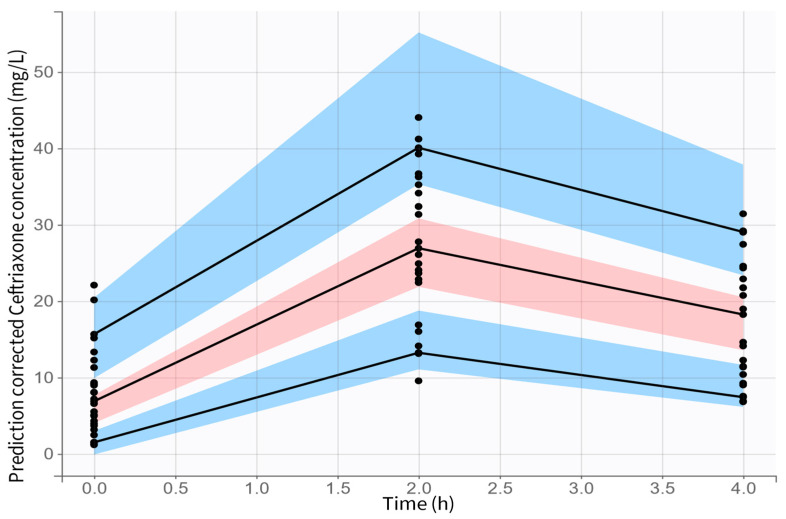
pcVPC plots for unbound ceftriaxone concentrations. Black points represent observed concentrations. Solid black lines represent the 10th, 50th, and 90th percentiles of observed concentrations. Colored areas represent the 95% confidence interval of the 10th and 90th (blue) and 90th (red) percentiles of simulated concentrations.

The inclusion of both covariates reduced the −2 LL and BIC by more than 23.53 and 10.82, respectively, with respect to the base model, indicating a statistically significant improvement upon adding the covariates in the final model (Figure 1). The model showed acceptable performance with good diagnostic plots and prediction-corrected Visual Predictive Check (pcVPC) (Figure 2).

### 2.3. Probability of Target Attainment

**Figure 3:**
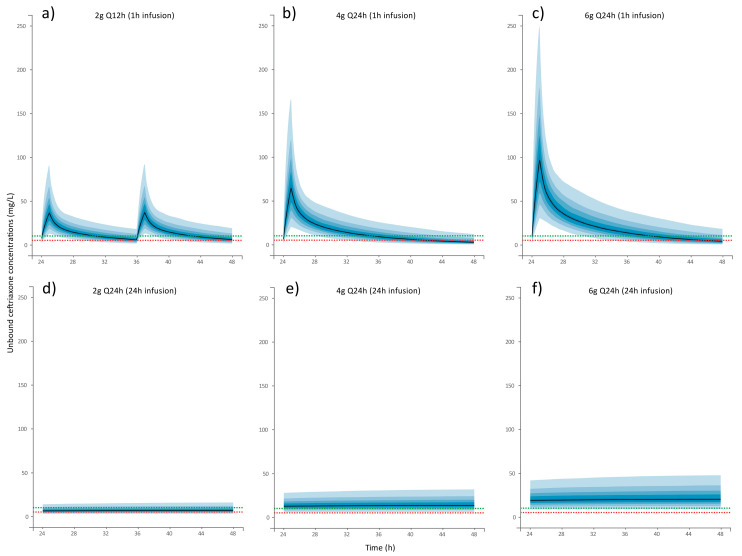
Simulated concentration profile (median and 5% percentiles) of ceftriaxone associated with dosing regimens of (**a**) 2 g Q12 h (1 h infusion) (**b**) 4 g Q24 h (1 h infusion), (**c**) 6 g Q24 h (1 h infusion), (**d**) 2 g Q24 h (24 h infusion), (**e**) 4 g Q24 h (24 h infusion), and (**f**) 6 g Q24 h (24 h infusion). Black lines represent median simulated concentrations. Blue gradient areas represent population 5% percentiles (5–95%). Red and green dashed lines denote for ceftriaxone concentrations of 5 mg/L and 10 mg/L (pharmacokinetic targets), respectively.

Figure 3 presents the Monte Carlo simulations of different ceftriaxone regimens and the PTAs for fT ≥ Cs ≥ 50%, 75% and 100% of the dosing interval, being the Cs target between 5 and 10 mg/L in Table 3. Regarding the administration of ceftriaxone in one-hour infusions, none of the dosage regimens maintained a Cs ≥ 10 mg/L for a minimum of 50% of the dose interval throughout the entire simulated population. Only the 2 g/12 h regimen achieved a fT > 5 mg/L for 50% of the interval dose in 90.6% of the simulated population. In contrast, the 4 g/24 h and 6 g/24 h regimens failed to reach the PK/PD targets.

On the other hand, continuous infusion increased pharmacodynamic target achievement. Simulations of 6 g/24 h in continuous infusion showed that >90% of the population would achieve unbound ceftriaxone concentrations ≥10 mg/L throughout the dose interval. Of the remaining doses tested, only 4 g/24 h maintained a Cs ≥ 5 mg/L for 100% of the dosing interval.

## 3. Discussion

Ceftriaxone is an antibiotic commonly used for severe infections. Despite the intrinsic resistance of *E*. *faecalis* to ceftriaxone, when it is combined with ampicillin its presence is essential to enhance ampicillin activity, improving clinical results when combating difficult-to-treat infections such as IE [4,6,10]. This study explored the pharmacokinetics of high-dose ceftriaxone in elderly patients. Our findings indicate that superior exposure is achieved when doses equal to or exceeding 4 g of ceftriaxone are administered only as a 24 h infusion.

Ceftriaxone pharmacokinetics has been previously studied. In recent years, research on this topic has focused on the pharmacokinetic alterations observed in critically ill adults and children, as well as the design of appropriate dose regimens [23,24,25,26,27,28,29,30]. In this population, the influence of ethnicity [31,32], augmented renal clearance [28], and extracorporeal therapies have been investigated [27,29]. Notably, the exposure goal in these studies vary between 50 and 100% of the 1–4 times above MIC (fT > MIC), leading to a C_min_ goal between 1 and 2 mg/L. Most studies agreed that maximal exposure could be achieved using continuous infusion of ceftriaxone [24,26,28,30]. In our study, the concentration target was higher (5–10 mg/L) to ensure the blockade of Penicillin Binding Proteins (PBP) 2 and 3 required to strengthen synergistic activity with aminopenicillins. Our results also indicate that continuous infusion is the optimal method for optimizing exposure, which also facilitates admission to OPAT programs.

Despite the growing interest in ceftriaxone pharmacokinetics in recent years, little has been explored regarding the influence of aging. A limited number of studies were conducted in the 1980s and 1990s [21,22,33] which failed to identify any significant differences in ceftriaxone pharmacokinetics between adult and elderly populations. However, some issues should be considered: first, in some studies, the subjects included were healthy individuals, and therefore, the influence of drug interactions, medical conditions, and deterioration on ceftriaxone pharmacokinetics were not examined. Second, dose regimens studied include single doses and low dose regimens (1 g per day), which might fail to identify the influence on ceftriaxone pharmacokinetics of plasma proteins binding saturation as a consequence of the administration of repeated high doses [21,22]. Furthermore, hypoalbuminemia is a common effect of the aging process that can be exacerbated by severe infections, which also impact ceftriaxone pharmacokinetics. One recent study analyzed ceftriaxone pharmacokinetics in elderly frail patients [34]. In that study, Tan et al. suggested that the estimation of drug clearance improved significantly when renal function was estimated based on cystatin-C level on this population. However, cystatin-C level is not included in routine analysis in most hospitals, which would hinder a wide application of this strategy. Our study considered both factors, by including only elderly patients with severe infections and multiple high-dose regimens. Therefore, our results will likely explain their influence on ceftriaxone distribution and elimination and, consequently, on global exposition.

Ceftriaxone exposure goals required to maintain synergy with ampicillin have not been well established. The initial research in the AC combination proposed a ceftriaxone concentration of 5–10 mg/L for optimum bacterial killing [15,35]. Based on those findings, the AC regimen was defined; however, a recent pharmacokinetic study demonstrated that these concentrations were not achieved when 2 g of ceftriaxone were administered every 12 h [19]. Lately, some studies have addressed this question [17,18,36]. Jimenez-Toro et al. used a murine model to propose two novel PK/PD indices previously proposed for β-lactam/β-lactamase inhibitor combinations [18]. However, these indices have not been defined in an endocarditis model. Therefore, its application in this scenario is hindered by the lack of consideration of essential aspects of this type of infection, such as the poor penetration in vegetation and biofilm of antibiotics, high bacterial loads in the site of infections or the presence of dormant bacteria. Due to the absence of validated novel PK/PD indices for *E. faecalis* infective endocarditis, and the information regarding ceftriaxone exposure obtained in individuals treated with the recommended inpatient dose regimen (2 g each 12 h), we accepted, as a clinically relevant PK/PD goal, 50 to 100% of time above a Cs of 5–10 mg/L. Our results show that ceftriaxone continuous infusion generates the best exposure profile to achieve synergistic free concentrations.

Our study presents several limitations. First, it was conducted with a relatively small sample size. This may be attributed to the low prevalence of IE caused by *E. faecalis*, which posed a challenge to patient inclusion. Nevertheless, we included a homogeneous and previously poorly studied population, which yielded valuable information regarding this group. Second, albumin plasma concentration was not recorded, despite being the primary source of ceftriaxone protein binding in plasma. Instead, total plasma protein was recorded. Third, the serum concentrations were employed as a surrogate for infection-site concentration. Nevertheless, the selection of a serum target was consistent with the exposure achieved with the gold standard treatment. In this regard, further studies may be necessary to analyze the exposure required at the infection site. Finally, the proposed dosing regimen was selected based on modeling and simulation approaches and requires confirmation in the clinical setting. Therefore, controlled trials will be necessary to establish the clinical efficacy of such therapeutic regimens.

In conclusion, ceftriaxone is necessary to ensure ampicillin-killing effect when treating *E. faecalis* IE. Our findings suggest that intermittent infusions could only attain the lowest PK/PD goal. However, optimal ceftriaxone exposure might be achieved using high-dose continuous infusions; therefore, this would be the preferred dose regimen in this scenario. These findings warrant further investigations on the impact of this strategy in the clinical outcomes.

## 4. Material and Methods

### 4.1. Study Design

A prospective observational pharmacokinetic study was conducted on a cohort of patients affected by *E. faecalis* IE caused by ampicillin-susceptible strains, attended in two tertiary teaching hospitals during 2021–2022.

### 4.2. Patients

Eligible participants were patients older than 55 years who were treated with high-dose ceftriaxone regimens (at least 4 g per day) for more than 48 h. Participants were excluded if they exhibited any of the following conditions: serum creatinine levels > 1.5 mg/dL or severe renal impairment (CrCl < 10 mL/min); active treatment with any drug that could interact with ceftriaxone; or a previous allergy to penicillin or cephalosporins. All participants provided written informed consent before their inclusion.

### 4.3. Data Collection

The following data were recorded from medical records: sex, age, weight, height, serum creatinine, total plasma proteins, diagnosed infection, and antibiotic treatment (including start date, drug, dose regimen, and hours of administration). Creatinine clearance standardized for 1.73 m^2^ was estimated using the Cockcroft–Gault equation [37]. The choice of treatment was at the discretion of the clinician.

Following successful inclusion in the study, three blood samples were extracted from each eligible patient at a steady state (at least 48 h after treatment start), at the following time-points: pre-dose (C_min_), two hours (2 ± 0.5) after the drug administration (C_2_), and four hours (4 ± 0.5) after the drug administration (C_4_). If one patient received more than one drug regimen, one set of samples was obtained from each regimen.

### 4.4. PK Sample Collection and Quantification of Plasma Ceftriaxone

At each time-point, blood samples were collected into EDTA-containing blood tubes and without delay inverted several times and centrifuged 10 min at 3000× *g*. After that, plasma was collected and stored at −80 °C until analysis. Ceftriaxone total and free plasma concentrations were measured by a liquid chromatography-tandem mass spectrometry (LC-MS/MS) (Agilent Technologies, Palo Alto, CA and AB Sciex, Darmstadt, Germany) assay using previously described and adapted to measure unbound concentrations [38]. The free ceftriaxone fraction was isolated by ultrafiltration at 37 °C using centrifugal devices (Amicon Ultra−0.5 mL 30 K, Millipore, Cork, Ireland). Total and unbound standard curves were linear over 5 to 1000 mg/L range and 0.5 to 200 mg/L, respectively. In both cases, accuracy and precision were 100% ± 15% and <15%, respectively. The lower limits of quantification were 3 and 0.5 mg/L for total and free ceftriaxone, respectively. The Food and Drug Administration (FDA) guidelines were followed in the validation process, and the results met the acceptance criteria.

*E. faecalis* isolation, identification and antimicrobial susceptibility determination was performed following standard procedures in both hospitals based on European Committee on Antimicrobial Susceptibility Testing (EUCAST) guidelines and recommendations.

### 4.5. Pharmacokinetics Analysis

Ceftriaxone time courses were analyzed using nonlinear mixed-effects modeling software (Monolix; 2024R1 version) and the stochastic approximation expectation maximization (SAEM) algorithm. Both one- and two-compartment structural models with first-order elimination were tested. The between-subject variability (BSV or ω) was ascribed to an exponential distribution. The residual variability (σ) was tested for additive, proportional, combined normal, or log-normal distributions.

Covariate model building was performed using sequential assessment of biologically plausible clinical parameters. Forward inclusion was based on the model selection criteria and significant correlation with one of the pharmacokinetic parameters. Creatinine clearance standardized for 1.73 m^2^ (CrCl), age, weight, body mass index (BMI), and sex were explored as covariates for each structural model. Continuous covariates were evaluated using log-linear relationships as follows:

$$\theta_{i}=\theta_{pop}\times {\left(\frac{{Cov}_{i}}{{Cov}_{median}}\right)}^{\beta_{cov}}$$

where *θ_i_* is the individual parameter estimate, *θ_pop_* is the typical population value, *Cov_i_* is the individual′s covariate value, *Cov_median_* is the median covariate value in the population (used for normalization), and *β_cov_* represents the estimated exponent describing the covariate′s effect.

The unique categorical covariate explored (sex), was performed as follows:

$$\theta_{i}=\theta_{pop}\times \left(\beta_{cov}\times {Cov}_{i}\right)$$

where sex was coded as a binary covariable (sex: 0 = female, 1 = male). Covariate selection was based on stepwise forward inclusion and backward deletion. Acceptance of a biologically plausible covariate requires a minimal −2 log likelihood (−2 LL) decrease of 3.84 units (chi-squared, 1 degree of freedom, *p* ≤ 0.05).

The Bayesian information criterion (BIC) was selected to test several hypotheses with regard to the final model, covariate effect on PK parameters, residual variability model, and structure of the variance-covariance matrix for the BSV parameters. PK parameters were properly estimated if the relative standard errors (RSEs) were inferior to 50%. The effect of a covariate was retained if it caused a decrease in the BIC and reduced the corresponding BSV. Visual inspection was used to evaluate the goodness-of-fit plots (observed-predicted concentration scatterplots and normalized prediction distribution error [NPDE] versus time-predicted concentration scatterplots) of each model. In order to evaluate the final model, 1000 Monte Carlo simulations per patient were performed to compute the prediction-corrected visual predictive check (pc-VPC) [39]. One thousand nonparametric bootstrap iterations with resampling of each population parameter were simulated and median (interquartile range [IQR]) values of each parameter were reported.

### 4.6. Simulations and Probability of Target Attainment

Monte Carlo simulations were performed using SIMULX (version 2024R1). Ceftriaxone dose regimens, including short and continuous infusions, were simulated as follows: Dosage 1, 2 g/12 h (1 h infusion); Dosage 2, 4 g/24 h (1 h infusion); Dosage 3, 6 g/24 h (1 h infusion); Dosage 4, 2 g/24 h (24 h infusion); Dosage 5, 4 g/24 h (24 h infusion); Dosage 6, 6 g/24 h (24 h infusion). For the probability of target attainment (PTA) analysis, an optimal Cs between 5 and 10 mg/L was considered. The PK/PD index to predict efficacy was defined as maintaining free concentrations superior to the Cs at least 50–100% of the dosing interval (fT ≥ Cs ≥ 50–100% of the dosing interval) [20]. Ceftriaxone doses were considered optimal if at least 90% of the simulated population was able to achieve the defined PK/PD targets.

### 4.7. Statistical Analysis

Categorical variables were summarized as percentages. Continuous variables were summarized as median and interquartile range (IQR) or mean and standard deviation (SD). The statistical analysis was performed using SPSS version 28.0 (SPSS Inc., Chicago, IL, USA) and R 3.6.1 software (https://www.r-project.org/).

## Figures and Tables

**Table 1:** Baseline demographic and clinical characteristics of patients (median and interquartile range).

Characteristics (Median [IQR]) ^a^	Cases (Total of 24 Episodes)
Males (%)	18 (75)
Age (years)	77 (71–78)
Weight (Kg)	90 (69.8–99.5)
Height (cm)	168 (161–180)
BMI (Kg/m^2^)	29.3 (26.7–33.3)
Serum protein (g/dL)	5.8 (5.5–6.5)
Serum creatinine (mg/dL)	1.1 (0.8–1.2)
CrCl (mL/min/1.73 m^2^)	59.5 (48.5–88.4)
Antibiotic treatment	
Ampicillin plus ceftriaxone (%)	24 (100%)
Ceftriaxone dosage	
2 g/12 h (%)	18 (75%)
4 g/24 h (%)	3 (12.5%)
6 g/24 h (%)	3 (12.5%)
Diagnosed infection	
*E. faecalis* infective endocarditis (%)	24 (100%)

^a^ All data are expressed as median [IRQ], except of those marked as “%” which are expressed as frequency.

**Table 2:** Parameter estimates of the final free ceftriaxone population pharmacokinetic model.

Parameter	Mean (%RSE)	Shrinkage (%)	Median (IQR) Bootstrap (N = 1000)
Cl (L/h)	11.57 (10.5)	0.51	11.33 (9.04–14.07)
Effect of CrCl on CL	0.62 (44.8)		0.5 (−0.32–1.1)
V_1_ (L)	43.6 (27.5)	14.16	33.9 (15.01–58.03)
Effect of BMI on V_1_	2.52 (37.3)		2.83 (−0.26–6.13)
Q (h^−1^)	19.8 (32.4)	26.38	20.97 (11.31–49.15)
V_2_ (L)	40.94 (12.2)	43.28	47.17 (39.94–58.67)
ω Cl	0.48 (15.9)		0.47 (0.31–0.6)
ω V_1_	0.77 (20.6)		0.82 (0.12–1.26)
ω Q	0.75 (40.8)		0.83 (0.33–1.38)
ω V_2_	0.09 (68.5)		0.12 (0.044–0.26)
σ	1.41 (20)		1.24 (0.5–1.95)

Cl, clearance; V_1,_ central volume; Q, intercompartmental clearance; V_2,_ peripheral volume; ω, coefficient of variation for between-subject variability; σ, constant error to ceftriaxone observations.

**Table 3:** Probability of target attainment (PTA) for a target defined as the percentage of the dose interval above a free ceftriaxone (fT) of 5 mg/L or 10 mg/L following different ceftriaxone dosing regimens. Green and red colors indicate a PTA ≥90% (success) and <90% (failure) of the simulated population, respectively.

Dose Regimen	Probability of Target Attainment					
	Target Cs = 5 mg/L			Target Cs = 10 mg/L		
	50% fT	75% fT	100% fT	50% fT	75% fT	100% fT
2 g/12 h (1 h infusion)	90.6	75.8	54.3	57.3	36.8	18.2
4 g/24 h (1 h infusion)	79.1	48.2	22.1	43.1	19.3	4.7
6 g/24 h (1 h infusion)	88.6	65.2	38	65.8	35.7	13.3
2 g/24 h (24 h infusion)	73.5	72	69.1	23.7	21.9	20
4 g/24 h (24 h infusion)	98	97.8	97.5	73.5	72	69.1
6 g/24 h (24 h infusion)	99.6	99.6	99.6	92	91.7	90.8

## Data Availability

The original contributions presented in this study are included in the article. Further inquiries can be directed to the corresponding author(s).
